# 2-Methoxy­phenyl 2-{2-[1-methyl-5-(4-methyl­benzo­yl)pyrrol-2-yl]acetamido}acetate

**DOI:** 10.1107/S1600536808020722

**Published:** 2008-07-09

**Authors:** Ben-Yong Lou, Xia Guo, Qi Lin

**Affiliations:** a Department of Chemistry and Chemical Engineering, Minjiang University, Fuzhou 350108, People’s Republic of China

## Abstract

The title compound, amtolmetin guacil, C_24_H_24_N_2_O_5_, is a new gastroprotective non-steroidal anti-inflammatory drug. In the crystal structure, the drug mol­ecule is linked into a one-dimensional structure along the *c* axis by weak N—H⋯O inter­actions between the amide groups. C—H⋯O and C—H⋯π inter­actions influence the packing.

## Related literature

For background, see: Tubaro *et al.* (2000 ▶); Vippagunta *et al.* (2001 ▶).
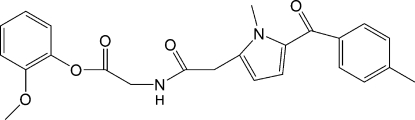


## Experimental

### 

#### Crystal data

C_24_H_24_N_2_O_5_
*M* *_r_* = 420.45
Orthorhombic, 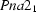

*a* = 11.307 (3) Å
*b* = 19.768 (7) Å
*c* = 9.713 (3) Å
*V* = 2170.9 (12) Å^3^
*Z* = 4
Mo *K*α radiation
μ = 0.09 mm^−1^
*T* = 293 (2) K
0.30 × 0.25 × 0.20 mm

#### Data collection

Rigaku Weissenberg IP diffractometer
Absorption correction: none
19812 measured reflections
2626 independent reflections
1938 reflections with *I* > 2σ(*I*)
*R*_int_ = 0.085

#### Refinement

*R*[*F* ^2^ > 2σ(*F* ^2^)] = 0.068
*wR*(*F* ^2^) = 0.109
*S* = 1.12
2626 reflections
284 parameters
1 restraint
H-atom parameters constrained
Δρ_max_ = 0.13 e Å^−3^
Δρ_min_ = −0.17 e Å^−3^

### 

Data collection: *TEXRAY* (Molecular Structure Corporation, 1999 ▶); cell refinement: *TEXRAY*; data reduction: *TEXSAN* (Molecular Structure Corporation, 1999 ▶); program(s) used to solve structure: *SHELXS97* (Sheldrick, 2008 ▶); program(s) used to refine structure: *SHELXL97* (Sheldrick, 2008 ▶); molecular graphics: *X-SEED* (Barbour, 2001 ▶); software used to prepare material for publication: *SHELXL97*.

## Supplementary Material

Crystal structure: contains datablocks I, global. DOI: 10.1107/S1600536808020722/bv2099sup1.cif

Structure factors: contains datablocks I. DOI: 10.1107/S1600536808020722/bv2099Isup2.hkl

Additional supplementary materials:  crystallographic information; 3D view; checkCIF report

## Figures and Tables

**Table 1:** Hydrogen-bonding geometry (Å, °) *Cg*1 is the centroid of the C2/C3–C7 ring.

*D*—H⋯*A*	*D*—H	H⋯*A*	*D*⋯*A*	*D*—H⋯*A*
N1—H1⋯O4^i^	0.88	2.32	3.1564	157
C11—H11*B*⋯O4^i^	0.99	2.40	3.2586	145
C24—H24*A*⋯O3^ii^	0.98	2.50	3.4418	162
C14—H14⋯ O1^iii^	0.95	2.43	3.3722	170
C19—H19⋯ O3^iii^	0.95	2.56	3.2300	127
C13—H13⋯ *Cg*1^iv^	0.95	2.85	3.6961	150

Symmetry codes: (i) 
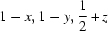
; (ii) 
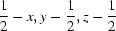
; (iii) 
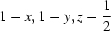
; (iv) 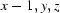.

**Fig. 1.**
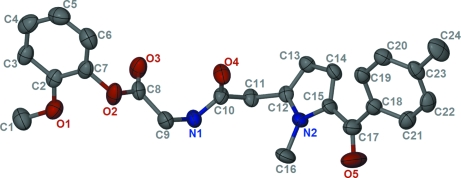
ORTEP of (I) with 50% thermal ellipsoids.

**Fig. 2.**
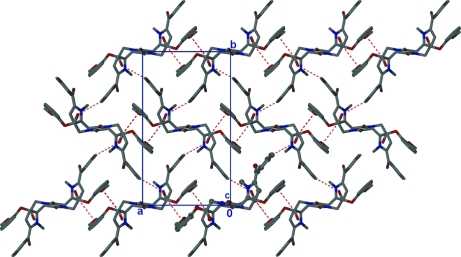
The packing structure viewed along c axis. The dashed lines indicate C—H···O or C—H···π interactions

## supplementary crystallographic information

### Comment

A current focus of research in solid state drug design is to understand polymorphism at the molecular level (Vippagunta *et al.*,  2001). Amtolmetin guacil is a new gastroprotective nonsteroidal anti-inflammatory drug (Tubaro *et al.*,  2000). In this contribution, we report its crystal structure which is unknown till now.

In the crystal structure, there exist weak hydrogen bonding interactions (N1—H1···O4) between the amide group of amtolmetin guacil which connect drug molecules into a one-dimensional structure along *c* axis. There also exist weak C—H···O interactions within the one-dimensional structure (C11—H11B···O4; C14—H14···O1; C19—H19··· O3; Table 1). The C—H···O interactions and C—H···π interactions (C24—H24A···O3; C13—H13···*Cg*1; Table 1) give rise to the packing structure (Fig.2)

### Experimental

Amtolmetin guacil (105 mg, 0.25 mmol) was dissolved in ethanol (20 ml) and the solution was kept in air and after several days colorless crystals were obtained.

### Refinement

All H atoms were located geometrically (C—H = 0.95–0.99 Å, N—H = 0.88 Å) with *U*_iso_(H) = 1.2 *U*_eq_(C,*N*) or 1.5 *U*_eq_(C).

### Crystal data

**Table:**

C_24_H_24_N_2_O_5_	*F*_000_ = 888
*M**_r_* = 420.45	*D*_x_ = 1.286 Mg m^−^^3^
Orthorhombic, *P**n**a*2_1_	Mo *K*α radiation λ = 0.71073 Å
Hall symbol: P2c-2n	Cell parameters from 19812 reflections
*a* = 11.307 (3) Å	θ = 3.5–27.5º
*b* = 19.768 (7) Å	µ = 0.09 mm^−^^1^
*c* = 9.713 (3) Å	*T* = 293 (2) K
*V* = 2170.9 (12) Å^3^	Block, colorless
*Z* = 4	0.30 × 0.25 × 0.20 mm

### Data collection

**Table:**

Rigaku Weissenberg IP diffractometer	1938 reflections with *I* > 2σ(*I*)
Radiation source: fine-focus sealed tube	*R*_int_ = 0.085
Monochromator: graphite	θ_max_ = 27.5º
*T* = 293(2) K	θ_min_ = 3.5º
scintillation counter scans	*h* = −14→14
Absorption correction: none	*k* = −25→25
19812 measured reflections	*l* = −12→12
2626 independent reflections	

### Refinement

**Table:**

Refinement on *F*^2^	Secondary atom site location: difference Fourier map
Least-squares matrix: full	Hydrogen site location: inferred from neighbouring sites
*R*[*F*^2^ > 2σ(*F*^2^)] = 0.068	H-atom parameters constrained
*wR*(*F*^2^) = 0.109	*w* = 1/[σ^2^(*F*_o_^2^) + (0.0337*P*)^2^ + 0.5426*P*] where *P* = (*F*_o_^2^ + 2*F*_c_^2^)/3
*S* = 1.12	(Δ/σ)_max_ < 0.001
2626 reflections	Δρ_max_ = 0.13 e Å^−^^3^
284 parameters	Δρ_min_ = −0.17 e Å^−^^3^
1 restraint	Extinction correction: SHELXL97 (Sheldrick, 2008), Fc^*^=kFc[1+0.001xFc^2^λ^3^/sin(2θ)]^-1/4^
Primary atom site location: structure-invariant direct methods	Extinction coefficient: 0.0111 (12)

### Special details

**Table:**

Geometry. All e.s.d.'s (except the e.s.d. in the dihedral angle between two l.s. planes) are estimated using the full covariance matrix. The cell e.s.d.'s are taken into account individually in the estimation of e.s.d.'s in distances, angles and torsion angles; correlations between e.s.d.'s in cell parameters are only used when they are defined by crystal symmetry. An approximate (isotropic) treatment of cell e.s.d.'s is used for estimating e.s.d.'s involving l.s. planes.
Refinement. Refinement of *F*^2^ against ALL reflections. The weighted *R*-factor *wR* and goodness of fit *S* are based on *F*^2^, conventional *R*-factors *R* are based on *F*, with *F* set to zero for negative *F*^2^. The threshold expression of *F*^2^ > σ(*F*^2^) is used only for calculating *R*-factors(gt) *etc*. and is not relevant to the choice of reflections for refinement. *R*-factors based on *F*^2^ are statistically about twice as large as those based on *F*, and *R*- factors based on ALL data will be even larger.

### Fractional atomic coordinates and isotropic or equivalent isotropic displacement parameters (Å^2^)

**Table:**

	*x*	*y*	*z*	*U*_iso_*/*U*_eq_	
O1	−0.0274 (2)	0.40855 (14)	0.2773 (3)	0.0591 (8)	
O2	0.1174 (2)	0.48242 (13)	0.4332 (4)	0.0637 (8)	
O3	0.2659 (2)	0.40913 (14)	0.4028 (4)	0.0647 (9)	
O4	0.4775 (2)	0.51742 (15)	0.4870 (3)	0.0595 (8)	
O5	0.7830 (4)	0.75559 (16)	0.3571 (4)	0.0995 (13)	
N1	0.4020 (2)	0.51066 (16)	0.2744 (3)	0.0481 (8)	
H1	0.4147	0.5028	0.1864	0.058*	
N2	0.6986 (2)	0.61548 (15)	0.3541 (3)	0.0438 (7)	
C1	−0.1080 (4)	0.3716 (3)	0.1921 (5)	0.0747 (14)	
H1A	−0.0888	0.3233	0.1960	0.112*	
H1B	−0.1016	0.3876	0.0969	0.112*	
H1C	−0.1890	0.3787	0.2252	0.112*	
C2	−0.0308 (3)	0.39568 (19)	0.4136 (5)	0.0469 (9)	
C3	−0.1055 (3)	0.35013 (19)	0.4785 (5)	0.0547 (11)	
H3	−0.1590	0.3239	0.4251	0.066*	
C4	−0.1032 (4)	0.3424 (2)	0.6190 (5)	0.0676 (13)	
H4	−0.1552	0.3111	0.6620	0.081*	
C5	−0.0266 (4)	0.3793 (3)	0.6973 (5)	0.0721 (13)	
H5	−0.0254	0.3735	0.7944	0.086*	
C6	0.0490 (4)	0.4250 (2)	0.6363 (5)	0.0678 (13)	
H6	0.1021	0.4509	0.6907	0.081*	
C7	0.0467 (3)	0.43263 (19)	0.4957 (5)	0.0505 (10)	
C8	0.2255 (3)	0.4638 (2)	0.3893 (4)	0.0464 (9)	
C9	0.2833 (3)	0.5239 (2)	0.3221 (4)	0.0538 (10)	
H9A	0.2344	0.5385	0.2429	0.065*	
H9B	0.2856	0.5618	0.3888	0.065*	
C10	0.4926 (3)	0.51018 (18)	0.3624 (4)	0.0448 (9)	
C11	0.6148 (3)	0.5008 (2)	0.3026 (4)	0.0478 (10)	
H11A	0.6407	0.4533	0.3153	0.057*	
H11B	0.6135	0.5106	0.2027	0.057*	
C12	0.6995 (3)	0.54758 (17)	0.3735 (4)	0.0402 (8)	
C13	0.7776 (3)	0.53269 (19)	0.4760 (4)	0.0456 (9)	
H13	0.7976	0.4888	0.5084	0.055*	
C14	0.8228 (3)	0.59397 (19)	0.5247 (4)	0.0486 (10)	
H14	0.8779	0.5993	0.5978	0.058*	
C15	0.7734 (3)	0.64510 (18)	0.4483 (4)	0.0458 (9)	
C16	0.6281 (4)	0.6504 (2)	0.2510 (4)	0.0677 (13)	
H16A	0.6737	0.6544	0.1656	0.102*	
H16B	0.6074	0.6956	0.2847	0.102*	
H16C	0.5556	0.6246	0.2332	0.102*	
C17	0.7972 (4)	0.7178 (2)	0.4555 (4)	0.0581 (11)	
C18	0.8422 (3)	0.74440 (19)	0.5879 (4)	0.0483 (9)	
C19	0.8074 (3)	0.71810 (18)	0.7124 (4)	0.0482 (10)	
H19	0.7539	0.6811	0.7145	0.058*	
C20	0.8493 (3)	0.74480 (19)	0.8353 (4)	0.0512 (10)	
H20	0.8244	0.7258	0.9204	0.061*	
C21	0.9197 (4)	0.79965 (19)	0.5888 (5)	0.0576 (11)	
H21	0.9435	0.8195	0.5042	0.069*	
C22	0.9615 (3)	0.8255 (2)	0.7100 (5)	0.0580 (11)	
H22	1.0152	0.8624	0.7080	0.070*	
C23	0.9273 (3)	0.79901 (19)	0.8349 (5)	0.0529 (10)	
C24	0.9719 (5)	0.8269 (2)	0.9692 (5)	0.0820 (16)	
H24A	1.0546	0.8408	0.9588	0.123*	
H24B	0.9662	0.7920	1.0406	0.123*	
H24C	0.9240	0.8661	0.9958	0.123*	

### Atomic displacement parameters (Å^2^)

**Table:**

	*U*^11^	*U*^22^	*U*^33^	*U*^12^	*U*^13^	*U*^23^
O1	0.0588 (17)	0.0615 (18)	0.0569 (18)	−0.0087 (14)	0.0102 (15)	−0.0022 (15)
O2	0.0390 (13)	0.0507 (15)	0.101 (2)	−0.0013 (12)	0.0066 (16)	−0.0015 (17)
O3	0.0476 (15)	0.0512 (17)	0.096 (2)	0.0026 (13)	0.0111 (16)	−0.0020 (16)
O4	0.0488 (15)	0.091 (2)	0.0383 (16)	−0.0072 (15)	0.0047 (13)	−0.0114 (16)
O5	0.164 (3)	0.065 (2)	0.069 (2)	−0.033 (2)	−0.042 (3)	0.0247 (19)
N1	0.0393 (17)	0.064 (2)	0.0406 (17)	−0.0050 (15)	−0.0003 (15)	−0.0036 (16)
N2	0.0472 (16)	0.0503 (18)	0.0339 (16)	−0.0026 (14)	−0.0076 (15)	0.0020 (15)
C1	0.058 (3)	0.102 (4)	0.064 (3)	−0.004 (3)	−0.003 (3)	−0.008 (3)
C2	0.040 (2)	0.043 (2)	0.057 (3)	0.0051 (17)	0.011 (2)	−0.0051 (19)
C3	0.048 (2)	0.043 (2)	0.073 (3)	−0.0034 (18)	0.012 (2)	−0.007 (2)
C4	0.064 (3)	0.063 (3)	0.076 (4)	0.006 (2)	0.024 (3)	0.012 (3)
C5	0.071 (3)	0.087 (3)	0.059 (3)	0.013 (3)	0.001 (3)	0.012 (3)
C6	0.052 (3)	0.079 (3)	0.072 (3)	0.002 (2)	−0.008 (2)	−0.009 (3)
C7	0.039 (2)	0.045 (2)	0.067 (3)	0.0008 (18)	0.007 (2)	0.001 (2)
C8	0.0365 (18)	0.050 (2)	0.052 (2)	−0.0055 (18)	−0.0036 (18)	−0.0102 (19)
C9	0.042 (2)	0.061 (2)	0.059 (3)	0.0005 (19)	−0.006 (2)	0.002 (2)
C10	0.046 (2)	0.050 (2)	0.039 (2)	−0.0093 (17)	0.0015 (19)	−0.0059 (19)
C11	0.045 (2)	0.062 (2)	0.036 (2)	−0.0069 (18)	−0.0002 (17)	−0.0103 (18)
C12	0.0370 (17)	0.050 (2)	0.0338 (19)	−0.0018 (16)	0.0052 (16)	−0.0062 (17)
C13	0.043 (2)	0.046 (2)	0.048 (2)	0.0054 (17)	−0.0036 (19)	−0.0019 (18)
C14	0.042 (2)	0.055 (2)	0.048 (2)	−0.0012 (18)	−0.0135 (18)	−0.002 (2)
C15	0.050 (2)	0.048 (2)	0.040 (2)	−0.0054 (17)	−0.0137 (19)	−0.0016 (18)
C16	0.077 (3)	0.071 (3)	0.055 (3)	−0.003 (2)	−0.029 (2)	0.015 (2)
C17	0.071 (3)	0.052 (2)	0.052 (3)	−0.008 (2)	−0.013 (2)	0.008 (2)
C18	0.053 (2)	0.038 (2)	0.054 (2)	−0.0030 (18)	−0.007 (2)	−0.001 (2)
C19	0.048 (2)	0.0395 (19)	0.058 (3)	−0.0020 (16)	−0.012 (2)	0.000 (2)
C20	0.057 (2)	0.046 (2)	0.051 (2)	0.0035 (19)	−0.007 (2)	−0.004 (2)
C21	0.060 (2)	0.043 (2)	0.070 (3)	−0.007 (2)	−0.002 (2)	0.003 (2)
C22	0.057 (2)	0.043 (2)	0.075 (3)	−0.0097 (18)	−0.004 (3)	−0.011 (2)
C23	0.052 (2)	0.043 (2)	0.063 (3)	0.0061 (18)	−0.021 (2)	−0.012 (2)
C24	0.098 (4)	0.069 (3)	0.079 (3)	−0.003 (3)	−0.034 (3)	−0.024 (3)

### Geometric parameters (Å, °)

**Table:**

O1—C2	1.349 (5)	C10—C11	1.511 (5)
O1—C1	1.431 (5)	C11—C12	1.499 (5)
O2—C8	1.346 (4)	C11—H11A	0.9900
O2—C7	1.406 (5)	C11—H11B	0.9900
O3—C8	1.181 (4)	C12—C13	1.363 (5)
O4—C10	1.230 (4)	C13—C14	1.397 (5)
O5—C17	1.223 (5)	C13—H13	0.9500
N1—C10	1.334 (5)	C14—C15	1.372 (5)
N1—C9	1.443 (4)	C14—H14	0.9500
N1—H1	0.8800	C15—C17	1.464 (5)
N2—C12	1.355 (4)	C16—H16A	0.9800
N2—C15	1.377 (4)	C16—H16B	0.9800
N2—C16	1.454 (5)	C16—H16C	0.9800
C1—H1A	0.9800	C17—C18	1.479 (6)
C1—H1B	0.9800	C18—C19	1.374 (5)
C1—H1C	0.9800	C18—C21	1.400 (5)
C2—C3	1.386 (6)	C19—C20	1.388 (6)
C2—C7	1.392 (6)	C19—H19	0.9500
C3—C4	1.374 (6)	C20—C23	1.388 (5)
C3—H3	0.9500	C20—H20	0.9500
C4—C5	1.365 (7)	C21—C22	1.367 (6)
C4—H4	0.9500	C21—H21	0.9500
C5—C6	1.377 (7)	C22—C23	1.376 (6)
C5—H5	0.9500	C22—H22	0.9500
C6—C7	1.374 (6)	C23—C24	1.504 (6)
C6—H6	0.9500	C24—H24A	0.9800
C8—C9	1.505 (5)	C24—H24B	0.9800
C9—H9A	0.9900	C24—H24C	0.9800
C9—H9B	0.9900		
			
C2—O1—C1	116.9 (3)	C10—C11—H11B	109.8
C8—O2—C7	117.5 (3)	H11A—C11—H11B	108.2
C10—N1—C9	120.6 (3)	N2—C12—C13	108.7 (3)
C10—N1—H1	119.7	N2—C12—C11	122.9 (3)
C9—N1—H1	119.7	C13—C12—C11	128.0 (3)
C12—N2—C15	108.9 (3)	C12—C13—C14	107.3 (3)
C12—N2—C16	124.7 (3)	C12—C13—H13	126.4
C15—N2—C16	126.4 (3)	C14—C13—H13	126.4
O1—C1—H1A	109.5	C15—C14—C13	107.9 (3)
O1—C1—H1B	109.5	C15—C14—H14	126.1
H1A—C1—H1B	109.5	C13—C14—H14	126.1
O1—C1—H1C	109.5	C14—C15—N2	107.2 (3)
H1A—C1—H1C	109.5	C14—C15—C17	128.5 (3)
H1B—C1—H1C	109.5	N2—C15—C17	124.2 (3)
O1—C2—C3	125.9 (4)	N2—C16—H16A	109.5
O1—C2—C7	116.4 (4)	N2—C16—H16B	109.5
C3—C2—C7	117.7 (4)	H16A—C16—H16B	109.5
C4—C3—C2	120.8 (4)	N2—C16—H16C	109.5
C4—C3—H3	119.6	H16A—C16—H16C	109.5
C2—C3—H3	119.6	H16B—C16—H16C	109.5
C5—C4—C3	120.4 (5)	O5—C17—C15	122.5 (4)
C5—C4—H4	119.8	O5—C17—C18	120.5 (4)
C3—C4—H4	119.8	C15—C17—C18	117.0 (3)
C4—C5—C6	120.3 (5)	C19—C18—C21	117.9 (4)
C4—C5—H5	119.9	C19—C18—C17	122.2 (3)
C6—C5—H5	119.9	C21—C18—C17	119.9 (4)
C7—C6—C5	119.3 (5)	C18—C19—C20	121.0 (3)
C7—C6—H6	120.4	C18—C19—H19	119.5
C5—C6—H6	120.4	C20—C19—H19	119.5
C6—C7—C2	121.5 (4)	C23—C20—C19	120.5 (4)
C6—C7—O2	119.7 (4)	C23—C20—H20	119.8
C2—C7—O2	118.6 (4)	C19—C20—H20	119.8
O3—C8—O2	124.5 (4)	C22—C21—C18	120.9 (4)
O3—C8—C9	127.1 (3)	C22—C21—H21	119.5
O2—C8—C9	108.4 (3)	C18—C21—H21	119.5
N1—C9—C8	113.5 (3)	C21—C22—C23	121.3 (4)
N1—C9—H9A	108.9	C21—C22—H22	119.4
C8—C9—H9A	108.9	C23—C22—H22	119.4
N1—C9—H9B	108.9	C22—C23—C20	118.4 (4)
C8—C9—H9B	108.9	C22—C23—C24	122.1 (4)
H9A—C9—H9B	107.7	C20—C23—C24	119.6 (4)
O4—C10—N1	121.5 (4)	C23—C24—H24A	109.5
O4—C10—C11	121.3 (3)	C23—C24—H24B	109.5
N1—C10—C11	117.2 (3)	H24A—C24—H24B	109.5
C12—C11—C10	109.4 (3)	C23—C24—H24C	109.5
C12—C11—H11A	109.8	H24A—C24—H24C	109.5
C10—C11—H11A	109.8	H24B—C24—H24C	109.5
C12—C11—H11B	109.8		

### Table 1 Hydrogen-bonding geometry (A%, °). Cg1 is the centroid of the C2/C3–C7 ring.

**Table:**

D—H···A	D—H (Å)	H···A (Å)	D···A (Å)	D—H···A (°)
N1—H1···O4^i^	0.88	2.32	3.1564	157
C11—H11B···O4^i^	0.99	2.40	3.2586	145
C24—H24A···O3^ii^	0.98	2.50	3.4418	162
C14—H14··· O1^iii^	0.95	2.43	3.3722	170
C19—H19··· O3^iii^	0.95	2.56	3.2300	127
C13—H13··· Cg1^iv^	0.95	2.85	3.6961	150

Symmetry codes: (i)  1-x,1-y,1/2+z; (ii) 1/2-x,y-1/2,z-1/2 (iii) 1-x,1-y,z-1/2; (iv) x-1,y, z.
